# Job strain and burnout in Spanish nurses during the COVID-19: resilience as a protective factor in a cross-sectional study

**DOI:** 10.1186/s12960-022-00776-3

**Published:** 2022-11-19

**Authors:** María del Mar Molero Jurado, África Martos Martínez, María del Carmen Pérez-Fuentes, Héctor Castiñeira López, José Jesús Gázquez Linares

**Affiliations:** 1 Department of Psychology, Faculty of Psychology, University of Almería, Almería, Spain; 2 Department of Psychology, Universidad Politécnica y Artística del Paraguay, Asunción, Paraguay; 3 12 de Octubre Hospital, Madrid, Spain; 4 Department of Psychology, Universidad Autónoma de Chile, Providencia, Chile

**Keywords:** Burnout, Resilience, Stress, Job satisfaction, COVID-19

## Abstract

**Background:**

Nurses are frequently exposed to chronic stress in the workplace generating harmful effects such as job strain and burnout. On the contrary, resilience has been shown to be a beneficial variable. The objective of this study was to analyze the relationship between dimensions of the Job Demand Control-Support model, resilience and burnout in nurses, and examine the mediating role of resilience between job strain and burnout.

**Methods:**

A descriptive, cross-sectional study reported in line with the STROBE guidelines. Active nurses were invited to complete an online questionnaire in September, 2020. With snowball sampling, 1013 nurses, with a mean age of 34.71, filled out the Job Content Questionnaire, the Maslach Burnout Inventory and the Resilience Scale.

**Results:**

The results showed the existence of four groups of professionals based on job strain. The nurses in the “High Strain” group (high demands and low control) showed higher scores in emotional exhaustion and cynicism, while those in the “Active Job” group scored higher in personal realization and resilience. The findings showed that job strain affects burnout in nurses, and this effect is mediated by resilience.

**Conclusions:**

The findings of this study showed that a high level of resilience could exert a fundamental role in ensuring well-being and proper job performance by nurses. Nursing managers should see to the personable variables or competencies that provide and favor an opportunity for nurses to widen and improve their practice, in pursuance of satisfying and responding better to people’s needs and the systems they work for.

## Introduction

Nurses are essential workers highly valued and recognized by society, and all the more so during the COVID-19 pandemic due to their importance in healthcare [1]. It cannot be ignored that during the work day nurses are exposed to chronic stress [2, 3], which generates harmful effects such as job strain, and even burnout [4–7]. Job stress and strain in nurses has increased with COVID-19 [8] and strong job strain has been linked to high number of individual and unit demands, generating an increase in fatigue by the end of the shift [9]. Nurse’s fatigue not only affects the health and well-being of the workers themselves and the organization due to absenteeism and nursing turnover [9, 10], but it is directly related to patient safety and care quality [11]. This study analyzed the relationship between job strain, which has hardly been evaluated in Spanish nurses, and burnout, and how strategies related to resilience can provide protection against negative effects.

## Background

This study was based on the “Job Demand-Control” model (JDC) [12, 13], which basically posits that job conditions linked to perceived demands and control of the job influence worker well-being. In this context, the situation of nurses in the context of the pandemic cannot be overlooked and research in protective strategies becomes even more necessary than ever.

Previous research has shown that the JDC-S (Job Demand Control-Support) model can predict worker exhaustion [14, 15]. Studies analyzing the situation of nurses during the pandemic have focused on this relationship. Along this line, the study by Butera et al. [16] found that the risk of ICU nurses developing burnout rose considerably during the pandemic due to the increase in workload and decrease in control and available resources. Similarly, Maglalang et al. [17] found that nurses with the highest risk of burnout were those subjected to the heaviest workloads, although this effect was buffered when workers perceived flexibility in their practice, and when they could control the length, tasks, time and location of their work.

Therefore, beyond the individual factors associated with burnout in nurses, this syndrome may also stem from organizational factors. The consequences of burnout, both direct for patients and workers and indirect at the organizational level, are high [18] and are of concern to the scientific community [19]. In particular, nurses with burnout show more symptoms of cognitive dysfunction, depression, sleep and metabolic alterations and worse quality of life [20, 21]. Burnout has negative effects on nurses’ work in that it reduces satisfaction, humanized care, care quality and patient safety, productivity and job commitment, and increases turnover [18, 22].

The resilience construct is based on positive psychology, and it refers to a process of positive adaptation, through which the individual returns to a state of well-being or performance (or even, surpasses it), in an adverse situation or context [23]. From the hedonic adaptation model of Graham and Oswald [24], adaptation refers to the human being's tendency to remain at a stable level of well-being, recovering when harmful events appear and getting used to good ones. Thus, when exogenous events threaten their well-being, people can recover if they manage to control the situation to a certain extent through the flow of cognitive and behavioral resources to cope with it [25]. Adversity in one’s job may be due to an isolated intense circumstance or less intense, but long-lasting and/or frequent, such as in healthcare crises and job stress [26]. Resilience is not innate or permanent, but can increase due to interaction between internal characteristics and external experience [27, 28]. The importance of this variable in the current labor context is made obvious by the need to respond to technological challenges, increased production demands, competitive pressure and flexible and adaptive organizational restructuring especially in highly stressful jobs [26, 28] such as nursing. In this context, resilience is a weighty positive resource when the individual works in a stressful variable workplace [29]. Thus, for example, during the early months of the COVID-19 healthcare crisis nurses were exposed to prolonged intense stress (organizational changes, conflicts, heavier workload, strong perceived threat to their own safety and to that of their loved ones) [30, 31], which they may often cope effectively with by building up resilience, due in part to job support received (both inside and outside the workplace) [32, 33]. The role of resilience in the negative effect of stress and the high demand to which nurses are exposed has been little studied and should not be overlooked, and this is the main reason for this study. Along this line, organizational support and active participation of nurses in policy-making or developing lines of action are essential to resilience, especially in worldwide healthcare crisis contexts, such as the COVID-19 pandemic [34]. Therefore, more studies are needed to elucidate the factors associated with resilience in nurses and identify the contexts where both personal and organizational intervention is necessary to improve well-being in nursing and optimum functioning of healthcare services.

In occupational healthcare, resilience has been shown to be a variable with desirable effects and especially helps to fight job burnout [35], tolerate high levels of stress in the workplace and increase engagement [36, 37] and a sense of job efficiency [38]. The review by Hartmann et al. [23] on its underlying mechanisms found that individual resilience has been shown to be a mediating variable in the relationship between resources and job demands with burnout and cynical attitude toward the job among other factors. However, insofar as we know, little is known or has been questioned on the role of resilience in the relationship between job strain and the burnout syndrome.

### Research aim

The objective of this study was to analyze the relationship between the dimensions of the Job Demand Control-Support model, resilience and burnout, examining the mediating role of resilience between job strain and burnout in nurses.

## Methods

### Research design

This study used a descriptive, cross-sectional research design, aimed at Spanish nurses. It was carried out after the first summer with restrictive measures due to COVID-19. We followed the Strengthening the Reporting of Observational Studies in Epidemiology (STROBE) Guidelines throughout the research process [39].

### Sample

The study population included nurses who voluntarily answered the questionnaires according to the following inclusion criteria: 1. Actively employed in the 6 months before and at time of survey. 2. Nurses in care services who were in direct contact with patients and their families. The nurses participating were performing their professional practice at the time of study in primary care and various specialized areas. Thus, the original sample was *N* = 1165, but after filtering the answers to control questions placed throughout the questionnaire cases were detected that had incongruent or random answers (specifically CQ1 discarded 66 cases, CQ2 59 cases and CQ3 27). Therefore, valid answers were received from 1013 participants (86.3%), leaving a study sample size of *N* = 1013 Spanish nurses. The mean participant age was 34.71 (*SD* = 9.35) of whom 88.05% (*n* = 892) were women.

### Procedure and data collection

Data collection began on September 7, 2020, during the early days of work and school activity after the first summer with restrictive measures due to COVID-19, and ended on September 28, 2020. The study ethics received approval and informed consent was given by the participants before collecting data. A CAWI (Computer Aided Web Interviewing) survey was used as access to healthcare institutions was restricted at the time it was implemented. The survey was spread on social networks through nursing WhatsApp groups at various hospitals and health centers. Scientific societies and nursing associations collaborated in diffusion of the Research Group’s website, and therefore, snowball sampling. The questionnaire took an estimated 15 min to complete. Participants were requested to answer honestly and were ensured the anonymity of their answers.

### Measures

*Demographic questionnaire* This form was developed by the study research team and included nine questions about age, sex, marital status, education and current occupational grade.

The validated Spanish version for nurses of the *Job Content Questionnaire* (JCQ) [40, 41] was used to evaluate job strain. This instrument based on the JDC-S model evaluates three dimensions (psychological demands, decision latitude and job support) on a Likert-type scale where answers are from 1 (totally disagree) to 4 (totally agree). The reliability found for the scale in this study was: for the total scale *ω* = 0.74; Psychological demands *ω* = 0.78; Job control *ω* = 0.78; Job support *ω* = 0.86.

The *Maslach Burnout Inventory-Survey* (MBI) [42], in its Spanish adaptation for human service professionals [43] was applied to measure burnout. It consists of 22 items evaluated on a seven-point Likert-type scale, in three dimensions: emotional exhaustion, depersonalization and personal accomplishment. The reliability indices found were: *ω* = 0.88 for the complete scale, *ω* = 0.91 for emotional exhaustion, *ω* = 0.64 in depersonalization and *ω* = 0.79 for personal accomplishment.

The Wagnild [44] *Resilience Scale* (ER-14), Spanish version by Sánchez-Teruel and Robles-Bello [45]. The scale has 14 items distributed in two factors: Personal Competence and Acceptance of oneself and of life. In this case, reliability was *ω* = 0.92 for the complete scale, *ω* = 0.91 for the personal competence factor and *ω* = 0.70 for acceptance of self and of life.

### Ethical considerations

This study was approved by the University of Almería Bioethics Committee (Ref: UALBIO2020/032). Participation was voluntary, and before starting to answer the questionnaire, the first page gave information related to the study and its purpose. The participants gave their consent by marking a box designated for the purpose, which then gave them access to the questionnaire. Anonymity was ensured in compliance with Organic Law 3/2018 of December 4th on Personal Information and Guarantee of Digital Rights. At all times, the study complied with the ethical principles consecrated in the Helsinki Declaration.

### Statistical analysis

The SPSS version 24.0 statistical package for Windows [46] was used for data processing and analysis. To determine the reliability of the evaluation instruments used, following the recommendations of Ventura-León and Caycho [47], the McDonald [48] omega coefficient was estimated.

High and low psychological demand and job control were determined by a cutoff point corresponding to the medians observed in the sample (*M*_d_ = 20 demand and *M*_d_ = 23 control), following the recommendations of Karasek [40]. Thus, the “high strain” profile was defined as a score over the median of psychological demands and a score below the job control median. Similarly, another three situations can be identified: “active jobs” (high psychological demand and high control), “passive jobs” (low psychological demand and low control), and “low strain” (low psychological demand and high control). To assign cases to the various job strain profiles, a two-stage cluster analysis was done that enabled categorical variables to be included (in this case demand and control, with the dichotomous option low/high). Then, to find any between-group differences in burnout and resilience, the Welch’s [49] ANOVA was applied with the Games–Howell post hoc test, since it did not meet the assumption of homogeneity of variances (Levene’s test *p* < 0.05). For estimating the effect size, in this case, the partial eta squared (*η*_*p*_^2^) was applied where < 0.01 irrelevant, 0.01 small, 0.06 medium, above 0.14 large. The correlation matrix between the study variables, mean scores and standard deviations were also found.

Finally, to test the relationships between variables and determine the possible mediating effect of resilience, a SEM was applied (Fig. 3). That is, a latent mediation model was computed with WLS (*weighted least squares*), specifying two impact paths of job strain (X) on burnout in nurses (Y): a direct effect and an indirect effect mediated by resilience (M), using the lavaan package [50] in JASP version 0.14 [51]. The following indices were used to evaluate model fit: the chi square/degrees of freedom ratio (*χ*^2^/*df*), which was considered optimum at < 3 [52, 53] and acceptable < 5 [54]; the CFI and GFI, which according to Hu and Bentler [54] must provide values > 0.95 to be considered optimum and > 0.90 for acceptable fit; and the RMSEA, which considers < 0.06 optimum and < 0.08 or very near acceptable. Mediation analysis has become a very popular focus in cross-sectional studies and a potential alternative to causal inference methods [55].

## Results

### Job stressors, burnout and resilience in nursing

Table 1 shows the correlation matrix of the study variables. Psychological demand correlated positively with emotional exhaustion and depersonalization. Furthermore, the control and support dimensions were negatively correlated with these two burnout factors and positively with personal accomplishment. Resilience was found to be positively associated with control and job support. However, no association was found with psychological demand.

**Table 1:** Job stressors, burnout and resilience

		Psychological demands	Job control	Job support	*M* (*SD*)
Emotional exhaustion	Pearson's *r*	0.397***	− 0.193***	− 0.285***	29.76 (12.33)
	Upper 95% CI	0.448	− 0.133	− 0.227	
	Lower 95% CI	0.344	− 0.251	− 0.340	
Depersonalization	Pearson's *r*	0.255***	− 0.184***	− 0.181***	9.56 (6.27)
	Upper 95% CI	0.312	− 0.124	− 0.121	
	Lower 95% CI	0.197	− 0.243	− 0.240	
Personal accomplishment	Pearson's r	0.024	0.425***	0.272***	37.57 (6.27)
	Upper 95% CI	0.085	0.475	0.329	
	Lower 95% CI	− 0.038	0.374	0.214	
Resilience	Pearson's r	0.038	0.226***	0.143***	79.74 (13.05)
	Upper 95% CI	0.100	0.284	0.203	
	Lower 95% CI	− 0.023	0.167	0.082	
	M (SD)	19.75 (3.13)	22.43 (3.30)	26.06 (5.23)	

Pearson’s correlations and descriptive statistics

***p < 0.001

**Fig. 1:**
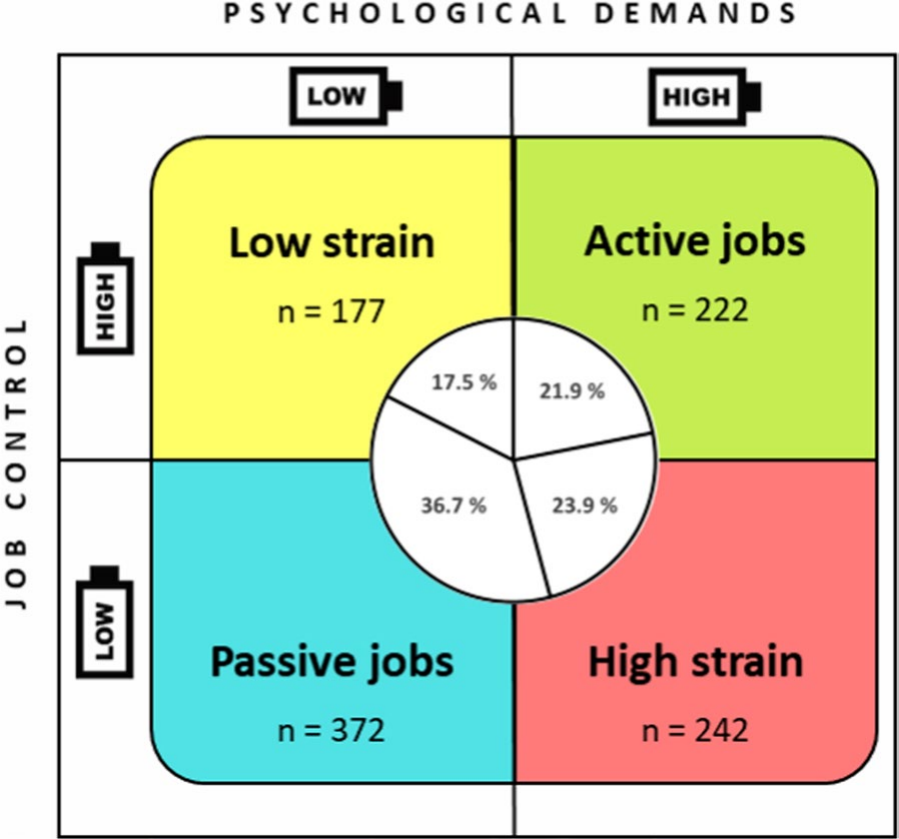
Classification of the sample into groups by job strain, following Karasek [36]

Figure 1 shows the distribution of the sample in the four job strain categories proposed by Karasek [36]. The median was used to find a dichotomy between high/low job control and high/low job demands: 23.9% were classified as high strain, 17.5% low strain, 36.7% were classified as passive jobs and 21.9% active jobs.

**Fig. 2:**
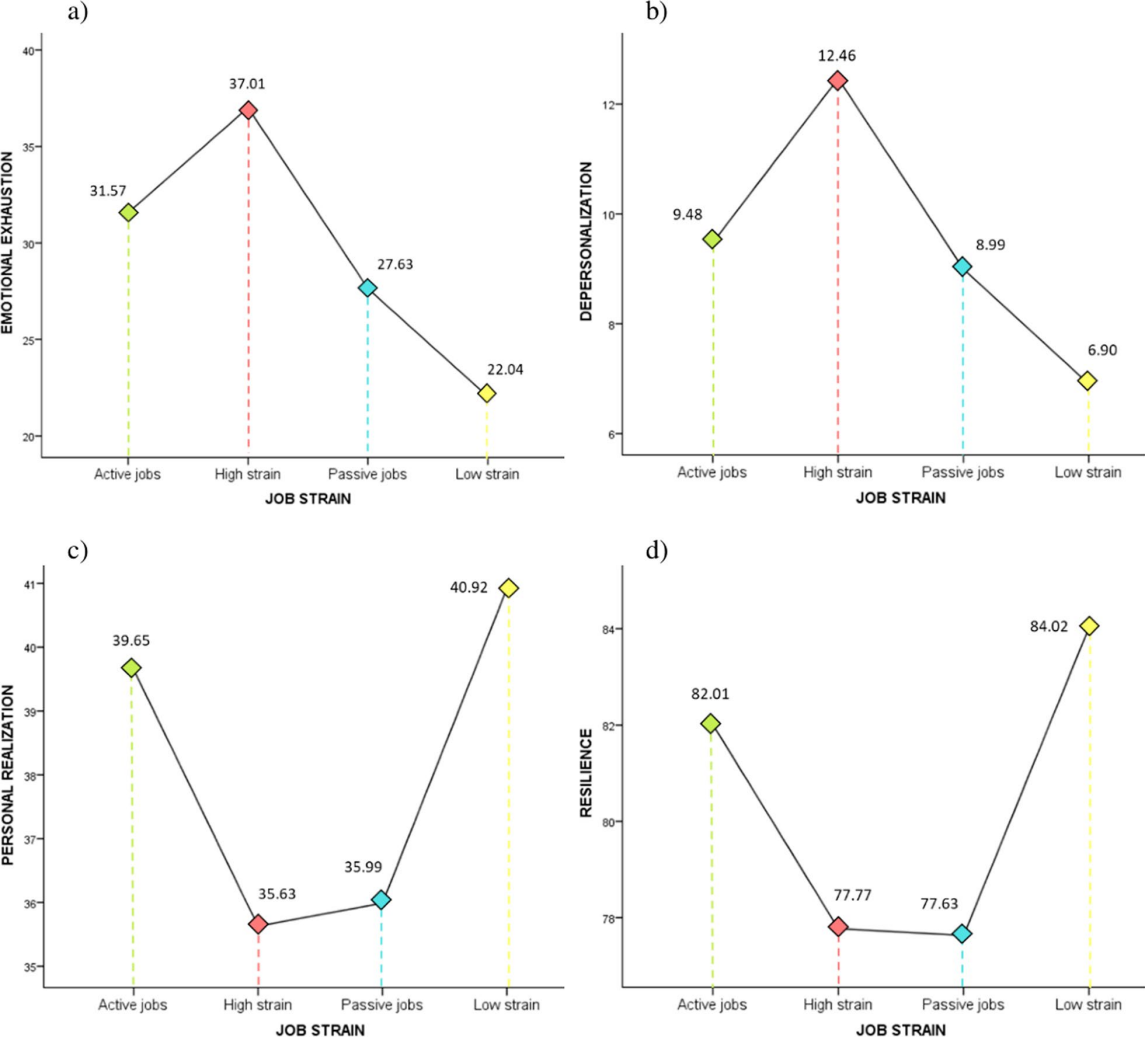
Burnout and resilience by job stress group. Mean plots **a** emotional exhaustion; **b** depersonalization; **c** personal realization; **d** resilience

However, multivariate analysis could not be used for between-group comparison of means, because when equality of covariance matrices were tested with Box’s M (*M*_Box_ = 112.28, *F* = 3.71, *p* < 0.001), the null hypothesis of equivalence of covariance matrices was rejected. The results of the univariate analysis for each dependent variable revealed between-group differences (see Fig. 2 for mean plots).

In the emotional exhaustion dimension (*F*_Welch_ = 74.75, *p* < 0.001, *η*_*p*_^2^ = 0.16), the high strain group had a significantly higher mean score than the rest of the groups.

There were also between-group differences in depersonalization (*F*_Welch_ = 37.29, *p* < 0.001, *η*_*p*_^2^ = 0.09), where the high strain profile again showed a higher score than the rest of the groups. In this dimension of burnout, active jobs did not differ significantly from the passive jobs group (*p* = 0.778).

Significant differences (*F*_Welch_ = 54, *p* < 0.001, *η*_*p*_^2^ = 0.12) were found in the personal accomplishment dimension of burnout between the active jobs and the high strain and passive job groups. There were no differences from the low strain group (*p* = 0.066).

Lastly, with respect to resilience, there were between-group differences (*F*_Welch_ = 16.06, *p* < 0.001, *η*_*p*_^2^ = 0.04) in the active job profile with significantly higher scores than in the high strain and passive job groups. On the contrary, the passive job group scored significantly lower in resilience than in the active job and low strain groups.

### The role of resilience with regard to job strain and nurse burnout

**Fig. 3:**
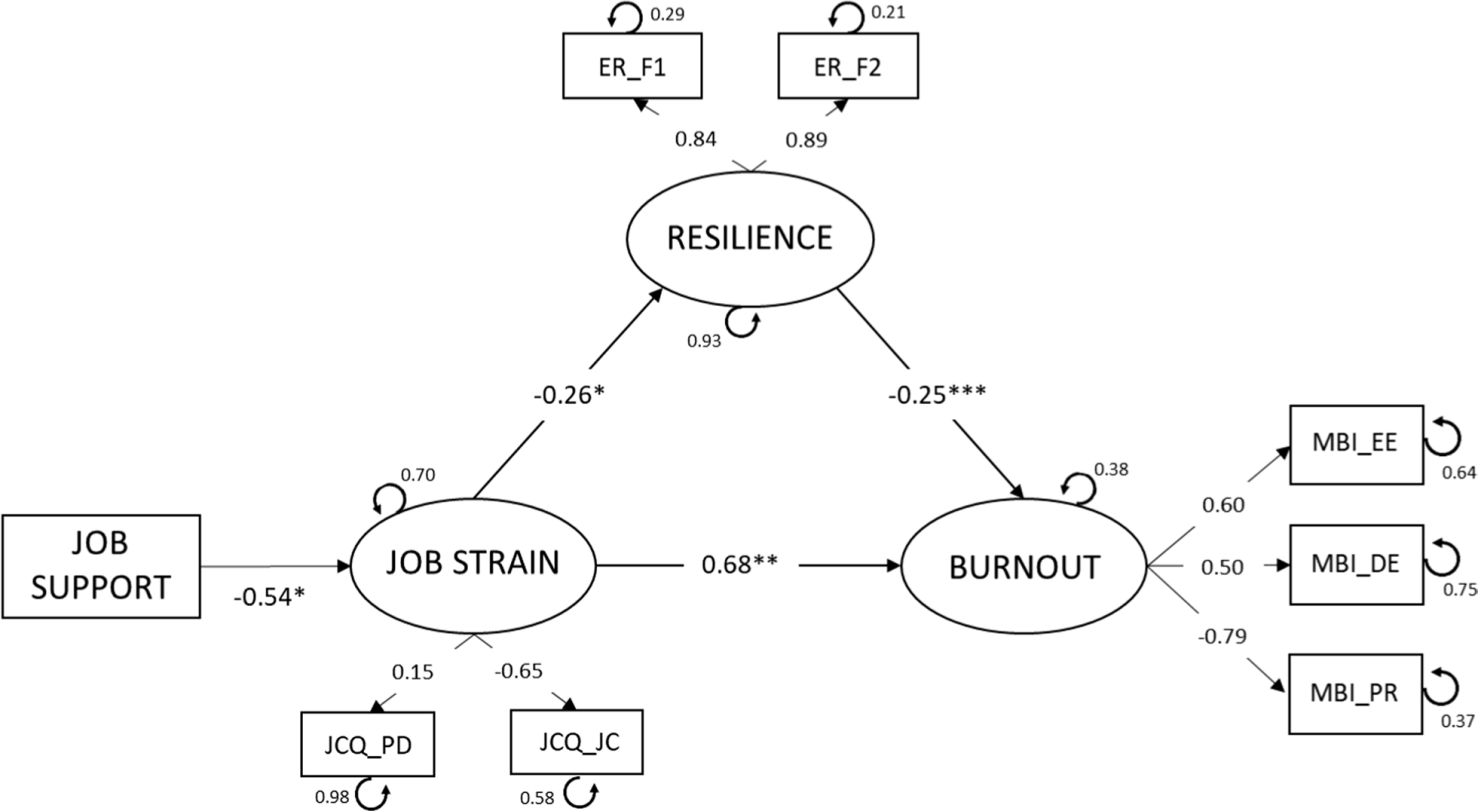
Structural equation model. Standardized parameters are shown. * *p* < 0.05, ** *p* < 0.01, *** *p* < 0.001. [JCQ_PD = psychological demands; JCQ_JC = job control; ER_F1 = personal competence; ER_F2 = acceptance of self and life; MBI_EE = emotional exhaustion; MBI_DE = depersonalization; MBI_PR = personal realization.]

The hypothesized model (Fig. 3) showed acceptable fit as indicated by: *χ*^2^ (13) = 55.18, *χ*^2^/df = 4.24, *p* < 0.001, CFI = 0.93, GFI = 0.95, RMSEA = 0.057 (ºCI 90% = 0.042, 0.072).

The relationships between the model variables were the following: job strain, influenced in turn by job support (− 0.54, *p* < 0.05), was negatively related to resilience (− 0.26, *p* < 0.05) and positively to burnout (0.68, *p* < 0.01). The relationship between resilience and burnout was negative (− 0.25, *p* < 0.001). In addition, in view of the total effect of job strain on burnout (0.75, *p* < 0.01) considering the magnitude of the indirect effect (0.06, *p* < 0.05), it may be concluded that the proportion (indirect/total) of this effect mediated by resilience is 0.039 to 0.133.

## Discussion

According to the results, the psychological demand and job strain dimensions are positively related to burnout’s emotional exhaustion and depersonalization; whereas the job control and social support dimensions are related negatively to these two burnout factors and positively to resilience and personal accomplishment. Thus, in line with previous studies [17], high job demand is linked to the presence of burnout in nurses.

Furthermore, nurses were clustered by their perception of demands and capacity for control in the workplace. The results showed the existence of four groups resulting from the combinations of high and low levels of the two dimensions mentioned above, as in the original Job Demand Control model [12, 13]. Nurses in the “Active Jobs” group (characterized by high levels of job control and demands) showed significantly higher levels of personal accomplishment and resilience. This may be because nurses who have the capacity to make decisions on how to cope with job demands and deploy their skills feel motivated and a sense of accomplishment in spite of a high workload. Thus, high demands in the healthcare sector would be perceived as a challenge and not discouraging. This motivation and decision-making capacity would help nurses to recover from extenuating work, which is an extremely important aspect of their job and should be kept in mind in managing nursing for planning training and job selection.

At the opposite end, are the nurses in the “Passive Job” group who have low levels of control and job demands. They scored significantly lower in resilience than the “Active Job” and “Low Strain” groups, which could be due to the lack of job pressure, which would not make recovery necessary. Nurses in the “High Strain” group corresponding to low control and high demands show significantly higher levels of emotional exhaustion and depersonalization. Finally, the group labeled “Low Strain”, with high control and low demands, were those with the highest resilience and personal accomplishment and lowest depersonalization and exhaustion, although these scores were not significantly different from those found in the “Active Job” group. In agreement with previous studies, we see how nurses who have higher scores in the burnout dimension are those with the highest workload, as long as they cannot perform their work flexibly and under their control, which has a buffering, and even, reparative effect [17, 56].

Finally, a model was tested to find the mediating effect of resilience in the relationship between job strain and burnout in nurses. According to the findings, social support by coworkers and supervisors reduces job strain. This strain in the workplace, in turn, affects nurses’ burnout levels and resilience acts as a mediator in this relationship. Thus, nurses exposed to high job strain would be less prone to develop burnout when they have high resilience. These results support previous evidence that shows the JDC model as a predictor of exhaustion [14, 15]. It should be emphasized that the mediating role of resilience reduces the negative impact of job strain in nurses. This is added to the evidence on positive intervention of this variable in palliating the effects of heavy workloads care professionals are exposed to [23].

### Implications for policy and practice

Nursing is one of the professions most exposed to high levels of sustained stress [2], especially since COVID-19 [30, 31]. This could easily lead to the appearance of job stress and even burnout [4]. Therefore, strong employee resilience can exert a fundamental role in ensuring well-being and proper job performance [23, 27, 36]. Some studies have shown that interventions for healthcare personnel should be designed to improve individual resilience after alleviating distress and improving the experience for future epidemics [57]. Increasing the psychological well-being and resilience of healthcare system workers, although already initiated, has become especially evident after COVID-19 [58].

### Future research

The saturation of healthcare systems and heavy employee workloads require study of factors that can counteract the negative effects of stress. Resilience has been identified as a beneficial variable, not only in nursing, but for healthcare workers in general [57, 58]. Therefore, in future, it would be of interest to corroborate whether these results may be applied to other healthcare professionals such as nursing aides or physicians.

Moreover, based on the Job Demands-Resources model it would be of interest to study the variables that could provide workers identified in the High Strain group with a stronger feeling of control. This could reduce burnout among the most saturated workers.

### Limitations

With respect to the limitations, we should highlight that the cross-sectional design of the study impeded establishing causal conclusions between job strain and burnout, which is important for interpreting the results of mediation. Although all the requirements for mediation analysis with a cross-sectional design were included in the study [49], we can only interpret the results in relational terms and encourage a future prospective study. Furthermore, the sample was collected from nurses actively employed and in different specializations and areas. This has two implications. On one hand, since one of the consequences of high job strain and burnout is quitting one’s job, it is possible that we did not have access to a sector of professionals especially affected by their work. On the other, not all the nursing units and specializations have the same capacity for control or emotional and physical workload. It would be of interest for future studies to use sampling techniques that provide access to the sector of the nursing population that left the profession. Also analyze possible repercussions of the specialization on the variables analyzed and whether all the nursing managers should consider them, regardless of the specialization they are in charge of. At this point, we must add that the type of sampling used in the study (snowball) has repercussions on the representativeness of Spanish nurses, and therefore the results may not be generalizable.

## Conclusions

Nursing is one of the professions most affected by job stress and work load. Even though they are highly valued as necessary professionals because of their contribution to health and population development, the consequences of frequent adverse situations to which they are subjected are many and remedies must be found to palliate them. The results of this study show that overloading these workers, without providing them with the capacity for controlling how to cope with the demands and without counting on job support, has consequences to their psychological well-being related to burnout. The findings show that job strain affects their burnout levels which are mediated by resilience.

Although it would be ideal for nursing management to give nurses better control in their job (more autonomy in decision-making, promotion of creativity and individual skills, etc.), or, at least, reorganize demands (distribute the number of tasks and requirements, and time pressure), it is also true that that is an arduous task involving integral change of the healthcare sector and populational education.

While these changes take place, resilience is a desirable variable that has been shown to mitigate the impact of job strain on burnout in nurses. Therefore, and since prior scientific evidence has shown that resilience as a variable that can be increased, it would be recommendable for nursing management to develop programs for improving the resilience of the nurses that they are in charge of. Additionally, and this is important, not only in the workplace itself, but also from earlier stages during their training. This would lead to improvement based on the evidence of their practice and health services, since by ensuring psychological well-being of the nurse, it promotes a general improvement in patient care. Full development of clinical competencies and nontechnical skills are necessary to strengthen the biopsychosocial process of recovery and care. Such demands particularly detected in nursing can and should be approached in other health sectors, analyzing them in other representative samples of professionals such as physicians, certified nursing aides, etc., after generalized action. Based on the comparison of results in different fields, it would be possible to identify differentiating variables among healthcare workers, adjusting the design of intervention to each case according to specific needs. Therefore, future lines of research should widen this type of study to other branches of healthcare, with attention to the characteristics of the professionals themselves (such as gender or personality) and also to the particularities of the job (performance of tasks, area, users, etc.).

## Data Availability

Data are available upon reasonable request to the corresponding author.
